# Increasing hernia size requires higher GRIP values for a biomechanically stable ventral hernia repair

**DOI:** 10.1016/j.amsu.2019.04.002

**Published:** 2019-04-19

**Authors:** F. Kallinowski, D. Gutjahr, M. Vollmer, F. Harder, R. Nessel

**Affiliations:** a Klinik für Allgemein-, Viszeral- und Transplantationschirurgie, Chirurgische Universitätsklinik Heidelberg, Im Neuenheimer Feld 110, D-69120, Heidelberg, Germany; b Technische Universität Hamburg-Harburg, Institut für Biomechanik, Eissendorferstrasse 38, D-21075, Hamburg, Germany; c Klinik und Poliklinik für Radiologie, Klinikum der Universität München, Marchioninistraße 15, 81377, München, Germany

**Keywords:** Ventral hernia repair, Grip, Bridging, Overlap, Fixation

## Abstract

**Background:**

Increasing hernia sizes lead to higher recurrence rates after ventral hernia repair. A better grip might reduce the failure rates.

**Material and methods:**

A biomechanical model delivering dynamic intermittent strain (DIS) was used to assess grip values at various hernia orifices. The model consists of a water-filled aluminium cylinder covered with tissues derived from pig bellies which are punched with a central defect varying in diameter. DIS was applied mimicking coughs lasting for up to 2 s with peak pressures between 180 and 220 mmHg and a plateau phase of 0.1 s. Ventral hernia repair was simulated with hernia meshes in the sublay position secured by tacks, glue or sutures as needed to achieve certain grip values. Grip was calculated taking into account the mesh: defect area ratio and the fixation strength. Data were assessed using non-parametric statistics.

**Results:**

Using a mesh classified as highly stable upon DIS testing (DIS class A) a reduced overlap without fixation led to early slippage (p < 0.001). With the application of 16 fixation points, transmural sutures were better than tacks with Securestrap^®^ being better than Absorbatack^®^ (p < 0.001). Plotting the likelihood of a durable repair as a function of the calculated grip higher grip values were needed with increasing hernia diameter to achieve biomechanical stability. This is important for clinical work since the calculated grip values both from a registry and from published data tend to drop as hernia sizes increase indicating biomechanical instability.

**Conclusion:**

The experimental work reported here demonstrates for the first time that higher grip values should be reached when repairing larger ventral hernias.

## Highlights

• Dynamesh^®^ Cicat safely bridges a hernia of 5 cms with a grip above 9.
• Increasing hernia size requires higher grip values.
• Hernia diameters of 10 cms require a grip above 50 using Dynamesh^®^ Cicat.
• Novafil^®^ sutures and Securestrap^®^ tacks provide better grip than Absorbatack^®^.
• In clinical work, grip values drop as hernia size increases.

## 1 Introduction

The incidence of incisional ventral hernias rises due to an ageing population experiencing higher rates of both obesity and major abdominal surgery [1]. Incisional hernias have a high recurrence rate related to site, patient condition and repair technique used [2]. Larger hernias recur more frequently with reported recurrence rates as high as 53% [3].

The high recurrence rates demand new ways to develop more stable ventral hernia repair procedures [4]. Coughs seem to rapidly impair ventral hernia repair [5]. A new bench test permits the analysis of biomechanical stability upon dynamic intermittent strain (DIS) which simulates coughing actions [6]. A dimensionless measure called “grip” can be derived which defines the durability of the reconstruction [7]. The grip of a repair takes into account the mesh: defect area ratio, the position of the mesh within the abdominal wall and the influence of a fixation technique [8]. In this manuscript, changes of the grip related to increasing hernia sizes are investigated.

## 2 Material and methods

**Fig. 1:**
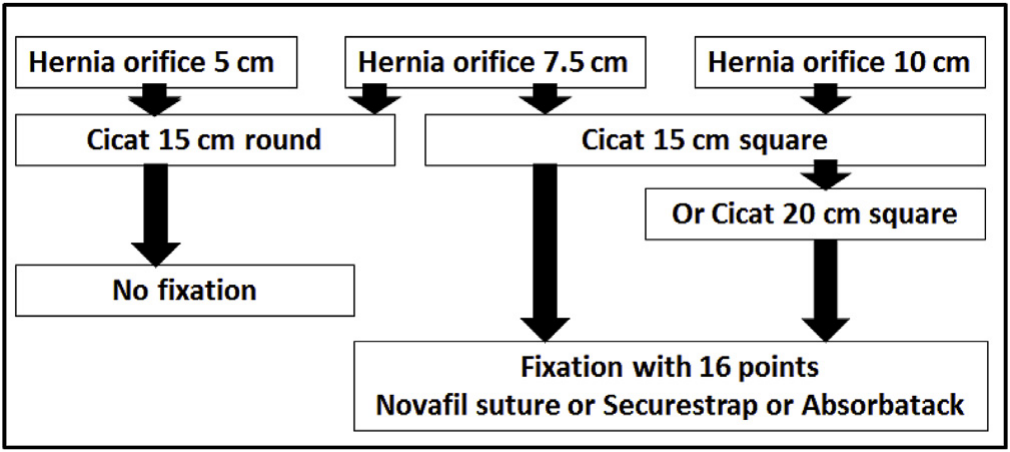
Experimental design used to analyze grip changes necessary to bridge different hernia sizes with biomechanically durable ventral hernia repair.

The DIS test has been described previously [[5], [6], [7], [8]]. In brief, the test bench consists of a water-filled aluminium cylinder coated with a thin polyethylene foil and covered with tissues derived from pig bellies. The tissues are punched with a central defect varying in diameter, bridged with a hernia mesh and loaded with cyclic impacts up to 220 mmHg. (for a depiction of the machine see http://www.hernie-heute.com/testverfahren/). The defect created in this study was always round and increased in size from 5 to 10 cms in steps of 2.5 cm (see Table 1). The defects were bridged in the sublay position with Dynamesh^®^ Cicat (FEG Textiltechnik, Aachen, Germany). The mesh was used either as a round or as a square material (Fig. 1). Since the mesh was previously classified as primarily stable (DIS class A) no fixation was necessary when used according to the instructions given by the manufacturer in proper sizes [8]. With reduced overlap and low mesh: defect area ratios, fixation was needed and applied with 16 points placed as a single crown. Novafil^®^ 2-0 sutures (Medtronic, Meerbusch, Germany), Securestraps^®^ (Ethicon, Norderstedt, Germany) and Absorbatacks^®^ (Medtronic, Meerbusch, Germany) were used for comparison to include strong and weak fixation methods [8].

**Table 1:** Conditions and basic statistical parameters of the experiments performed.

Defect size	Mesh size	Mesh shape	Fixation	Mean	Standard error	Minimum	25% quartile	Median	75% quartile	Maximum	Depicted in
(cm)	(cm)		16 points								
5	15	round	none	425	0	425	425	425	425	425	Fig. 3
7.5	15	round	none	14	6	7	10	12	19.25	23	Fig. 3
7.5	15	square	Securestrap	425	0	425	425	425	425	425	Fig. 4
7.5	15	square	Absorbatack	352	111	134	277.75	419	425	425	Fig. 4
10	15	square	Novafil	384	131	12	425	425	425	425	Fig. 5
10	15	square	Securestrap	127	155	2	21.75	46.6	204.5	425	Fig. 5
10	15	square	Absorbatack	18	25	1	2	7	21	66	Fig. 5
10	15	square	Novafil	425	0	425	425	425	425	425	Fig. 6
10	15	square	Absorbatack	229	174	6	52.75	249.5	402.25	425	Fig. 6

In this study, DIS was applied mimicking coughs lasting for up to 2 s with peak pressures between 180 and 220 mmHg and a plateau phase of 0.1 s. Ventral hernia repair was simulated with hernia meshes in the sublay position secured by tacks, glue or sutures as needed to achieve certain grip values. Grip of the reconstructions was calculated taking into account the mesh: defect area ratio (MDAR) and the fixation strength as described earlier [8]. Data were assessed using non-parametric statistics as described earlier [[6], [7], [8]].

## 3 Results

**Fig. 2:**
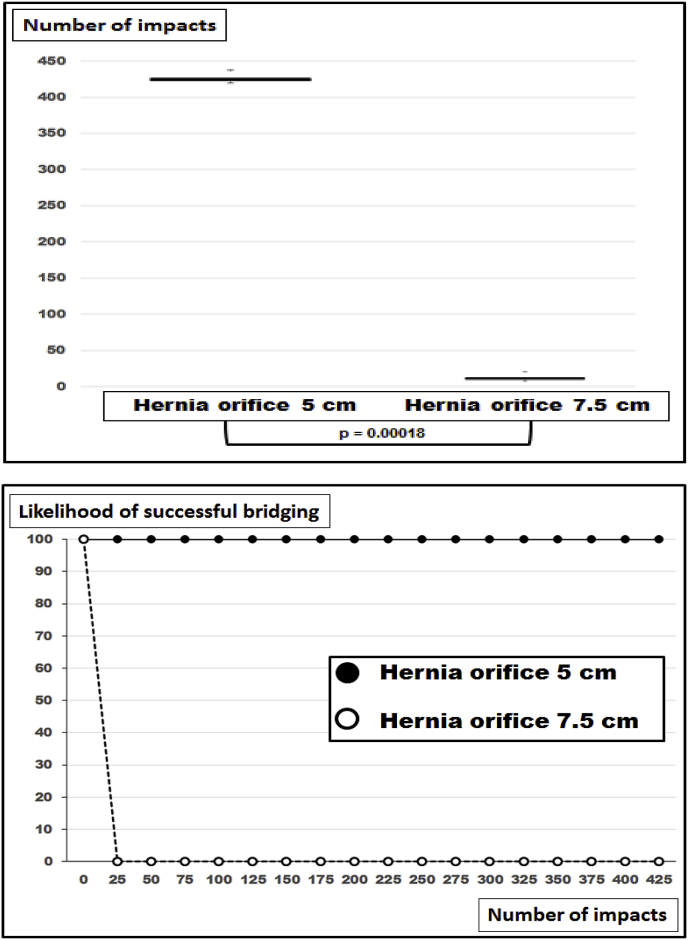
Box-and-whisker-plots (top) and likelihood curves (bottom) of ventral hernia repairs bridging hernia defects of two diameters with Dynamesh^®^ Cicat 15 cm round without fixation. P denotes statistically significant differences in the u-test.

Without fixation, Dynamesh^®^ Cicat with a diameter of 15 cm bridges a 5 cm round defect safely with no dislocation occurring upon 425 DIS impacts (Fig. 2). Under these conditions, MDAR as a measure of the grip can be calculated as 9 according to Tulloh and de Beaux [9]. Increasing the defect to a diameter of 7.5 cm lead to dislocation to occur in each specimen before the 25th DIS cycle (median: 12; range: 7–23 impacts, Fig. 2). MDAR as a measure of the grip more than halved to about 4 with increasing defect size in this experiment.

**Fig. 3:**
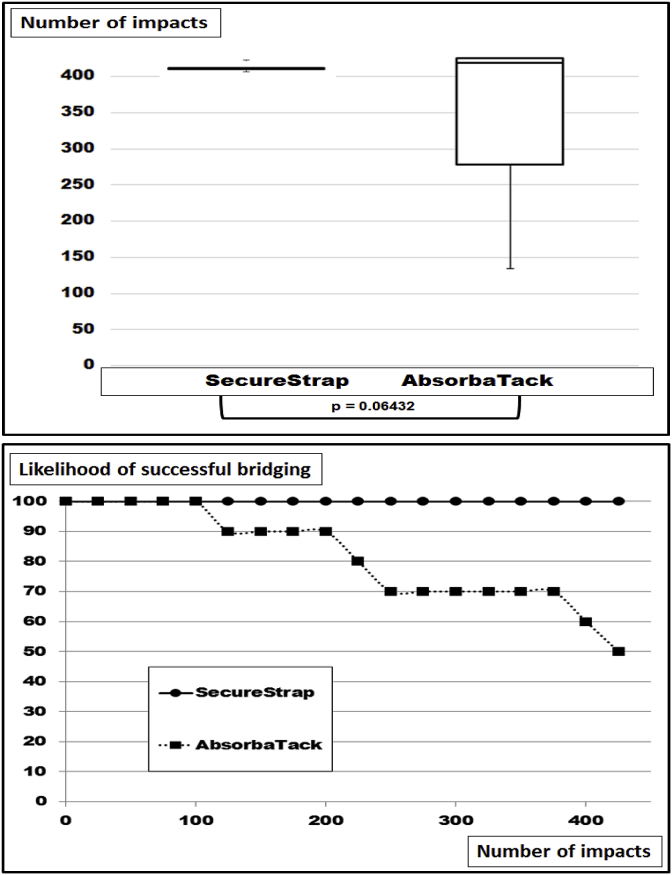
Box-and-whisker-plots (top) and likelihood curves (bottom) of ventral hernia repairs bridging hernia defects with a diameter of 7.5 cm with Dynamesh^®^ Cicat 15 cm square with 16 point of fixation using Securestrap^®^ as a strong and Absorbatack^®^ as a weak fixation device. The differences were not found to be significant upon u-testing.

A hernia size of 7.5 cm was bridged with a 15 cm square Dynamesh^®^ Cicat fastened with 16 points 1 cm inward from the edges. Securestrap^®^ was used as a strong fixation device according to Kallinowski et al., 2018 [8]. With this setup, 100% safety levels were reached again (Fig. 3). Grip can be estimated as being 41 under these conditions [8]. With 16 points of Absorbatack^®^ as a weak fixative placed as mentioned above similar safety levels were reached for the first 100 DIS impacts (Fig. 3). Thereafter, dislocation occurred leaving half of the reconstructions displaced after 425 strains (median DIS impacts at dislocation: 419; range: 134–425). Grip was calculated to be 27 in this experiment. Upon u-testing, the trend to dislocation was not found to be statistically significant.

**Fig. 4:**
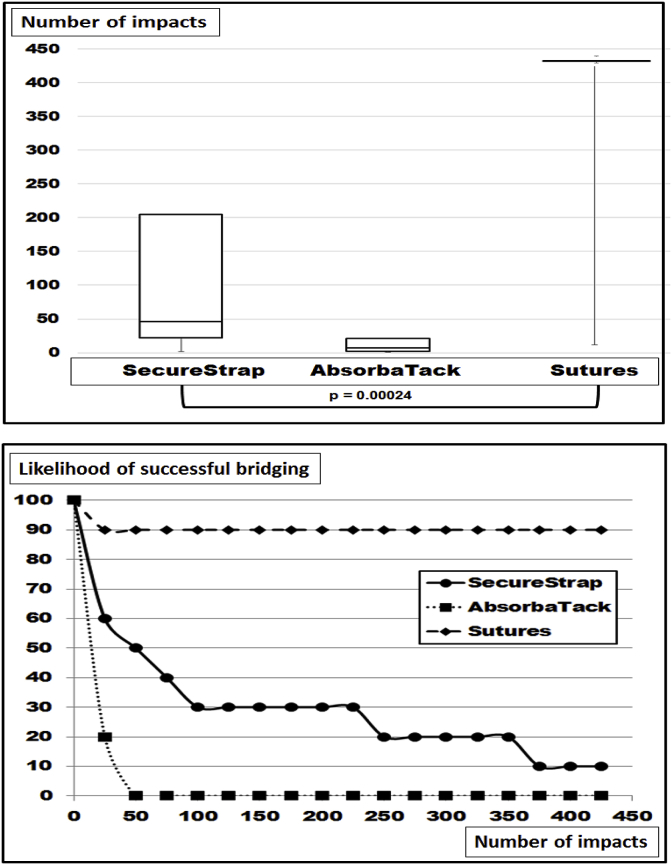
Box-and-whisker-plots (top) and likelihood curves (bottom) of ventral hernia repairs bridging hernia defects with a diameter of 10 cm with Dynamesh^®^ Cicat 15 cm square with 16 point of fixation using Novafil^®^ sutures or Securestrap^®^ as a strong and Absorbatack^®^ as a weak tacking device. The differences were found to be statistically significant with the Kruskal-Wallis-test.

Increasing the hernia size to 10 cm and leaving the mesh at 15 cm square dislocation was observed in all groups (Fig. 4). Distinctly different curves characterizing the various fixation techniques were observed (Fig. 4 bottom). The differences were highly significant with the Kruskal-Wallis test (p = 0.00024). The best fixation was provided by 16 transmurally placed Novafil^®^ sutures giving way only once. In this case early dislocation occurred at the 12th DIS impact due to one suture giving way without any obvious reason. Fixation to withstand 425 DIS impacts was observed in 9 out of 10 preparations. With the use of 16 Securestraps^®^ a gradual loss of fixation was observed starting early with the 2nd DIS impact. Only one repair was able to take 425 DIS impacts without dislocation. In the median dislocation occurred after the 46th DIS impact. Using Absorbatack^®^ with 16 fixation spots, dislocation occurred early in all preparations (median: 7; range: 1–66). Calculating the grip according to Kallinowski et al. [8] values well above 22 were reached with Novafil^®^ and Securestrap^®^. Lower grip levels just above 15 were reached with Absorbatacks^®^.

**Fig. 5:**
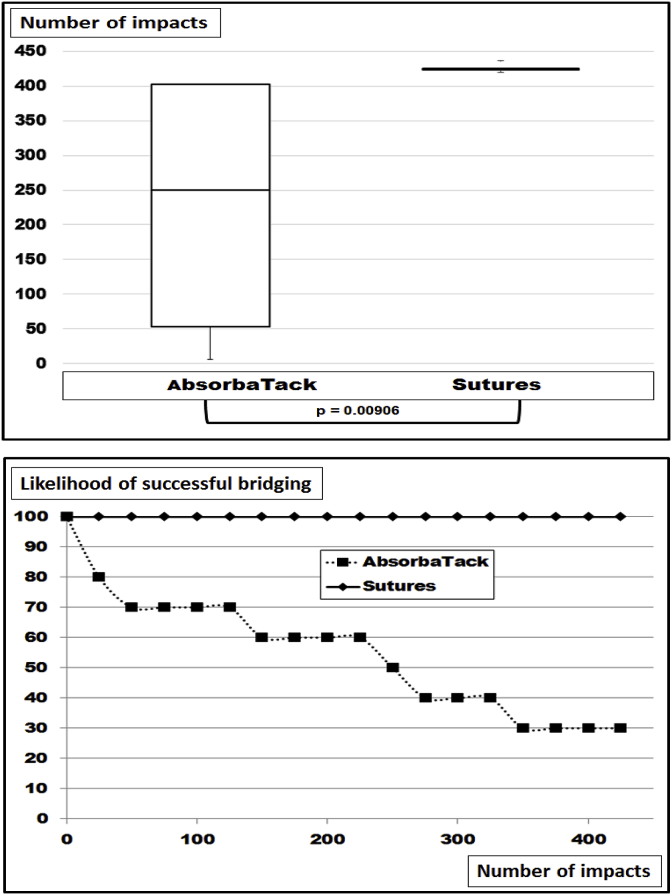
Box-and-whisker-plots (top) and likelihood curves (bottom) of ventral hernia repairs bridging hernia defects with a diameter of 10 cm with Dynamesh^®^ Cicat 20 cm square with 16 point of fixation using Novafil^®^ sutures as a strong fixation and Absorbatack^®^ as a weak tacking device. The differences were found to be statistically significant with the Mann-Whitney-u-test.

Leaving the hernia diameter at 10 cm and increasing the mesh size to 20 cm square improved the fixation strength of the repair to hold 425 DIS impacts in each of the 10 repetitions when 16 Novafil^®^ sutures were used for fastening (Fig. 5). With the use of 16 Absorbatack^®^ seven out of 10 reconstructions failed eventually yielding a significantly lower safety level of 30% after 425 DIS impacts (p = 0.00906). The calculation of grip levels yielded a figure of almost 41 for 16 Novafil^®^ sutures and about 27 for 16 Absorbatack^®^ under these conditions.

## 4 Discussion

Technically speaking is ventral hernia repair a compound technique. Tissue and textiles are bound together by stiction. The threshold for delamination depends on the mesh: defect area ratio and the fixation strength reached by sutures, tacks or glue [8]. This threshold can be summed up in a grip factor characterizing each individual reconstruction [8]. Due to high recurrence rates and rising socioeconomic cost, ventral hernia repair requires better standardization [1]. Considering biomechanical stability as a prerequisite for a stable scar formation there is a need for mechanical testing of reconstructions [4]. Efforts have been made to calculate the contribution of the meshes, the implantation procedures and of the fixation methods [10]. Calculation of the grip factor derived from DIS testing is a novel way to reach clinically relevant conclusions [8]. Hernia size has the potential to strongly influence mechanical stability and is investigated here.

**Fig. 6:**
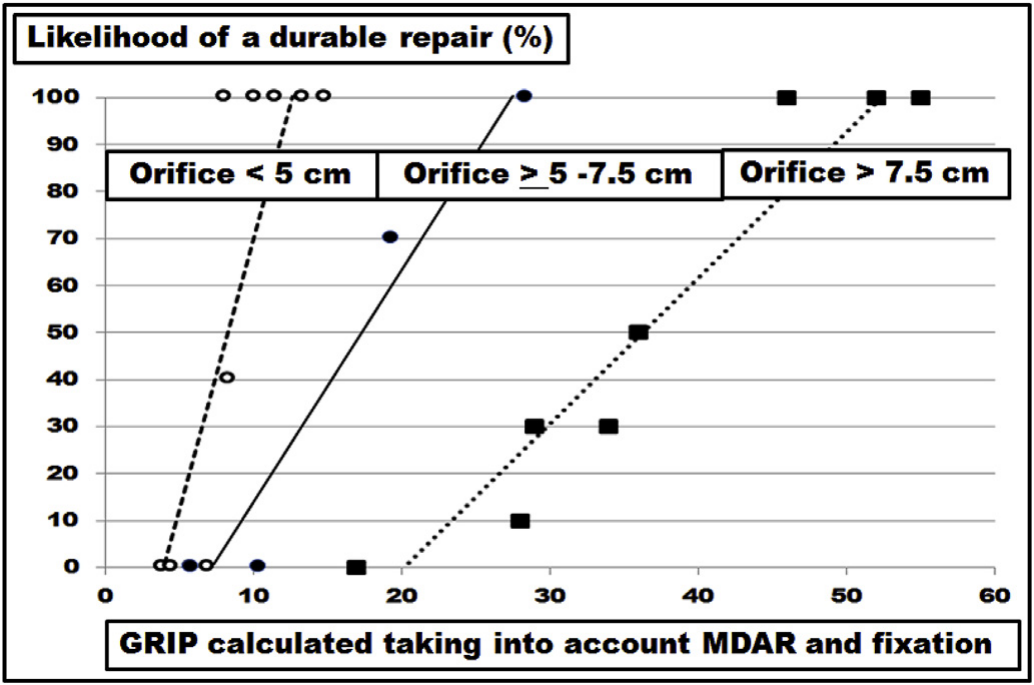
Likelihood for a durable repair surviving 425 DIS impacts as a function of the grip calculated as previously described [8]. The differences of the grip necessary to durably bridge various diameters were statistically significant with the Kruskal-Wallis-test (p = 0.00042). Data for hernia diameters up to 7.5 cm from previous publications [7,8] were included. The lines indicate the trend.

With submaximal load no failure of the mesh-tissue-interface is observed with increasing pressures below a threshold of about 150 mmHg [4,11]. In patients, up to 400 coughs were observed within 24 h which can reach intraabdominal pressures well above 200 mmHg [12,13]. Using a self-built device delivering dynamic impact strain (DIS) simulating coughing actions, repeated impacts can rapidly impair ventral hernia repair [5,6]. The grip calculation can give the threshold for a repair to survive more than 400 DIS strains [7,8]. From the data presented previously a grip factor was calculated for ventral hernia diameters of 5 and 7.5 cm varying between 6 and 24 dependent on the meshes used [8]. Combining the data reported previously with the new experiments detailed here for Dynamesh^®^ Cicat only, a size-dependent assessment is possible for this hernia mesh (Fig. 6). As hernia diameter increases from 5 to 7.5 cm, the grip necessary for a repair to sustain 425 DIS impacts increases from 10 to 20. With the hernia diameter measuring above 7.5 cm up to 10 cm, the grip necessary for a safe repair increases to a median of 50 (range: 46–55). With the Kruskal-Wallis-test, the necessary increase is statistically significant (p = 0.00042). Most randomized controlled studies quote hernia dimensions as an inclusion criteria but fail to report the mesh sizes used in relation to the hernia size [1]. Hernia overlap not tailored to the diameter of the hernia orifice is recognized as a key determinant of hernia recurrence [14,15]. Calculating the grip can take into account the mesh-defect area ratio and might give a unifying view on the various types of repair [8,9]. As a rule of thumb, the grip derived from a hernia diameter of 5 cm may be taken 2.5fold for an orifice of 7.5 cm and 5fold for a 10 cm wide hernia. At this point in time, this rule is based on Dynamesh^®^ Cicat only.

There are no systematic reviews or meta-analyses in the literature where different sizes of the hernia defect have been assessed [16]. In a recent Cochrane review, only three out of seven studies for open repair of incisional hernias addressed differences in sizes at all [17]. Experimentally, the size of a defect was the most influencial parameter to assess the stress of the repair upon strain [18]. Since meshes differ in their material and in their structure, the biomechanical response to strain and the durability of a repair differ from mesh to mesh. Classifying meshes taking the durability towards repeated dynamic strain into account provides a three-level classification [8]. Lower DIS classes need more overlap and/or more fixation for a biomechanically durable repair. Larger hernia sizes require even larger hernia meshes or more fixation points as demonstrated here (Fig. 2, Fig. 3, Fig. 4, Fig. 5, Fig. 6).

**Fig. 7:**
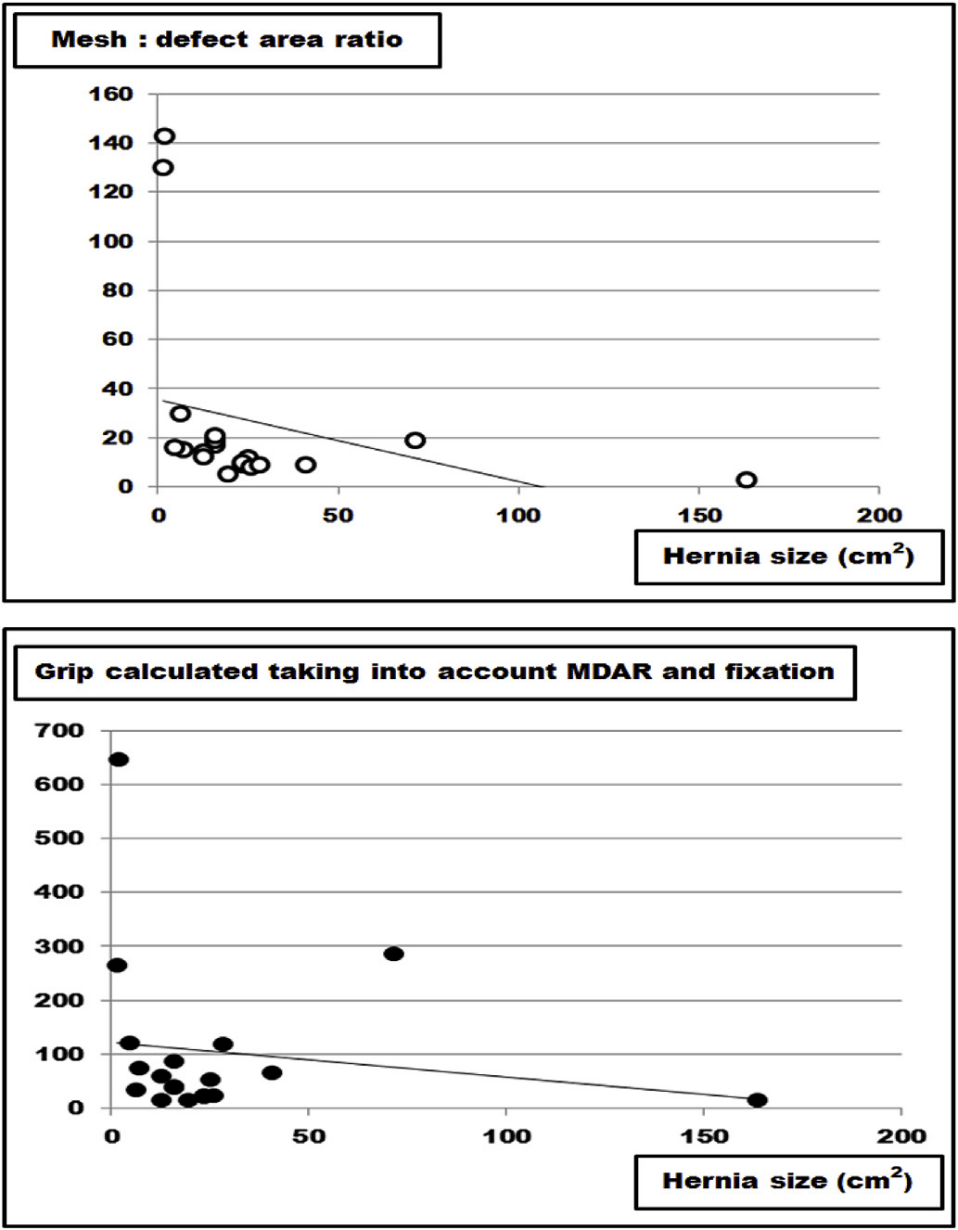
Data from the Stronghold application of the Herniamed registry as reported earlier [8]. It is obvious that both the mesh: defect area ratio (MDAR) and the grip drop as hernia sizes increase. The line indicates the trend.

**Fig. 8:**
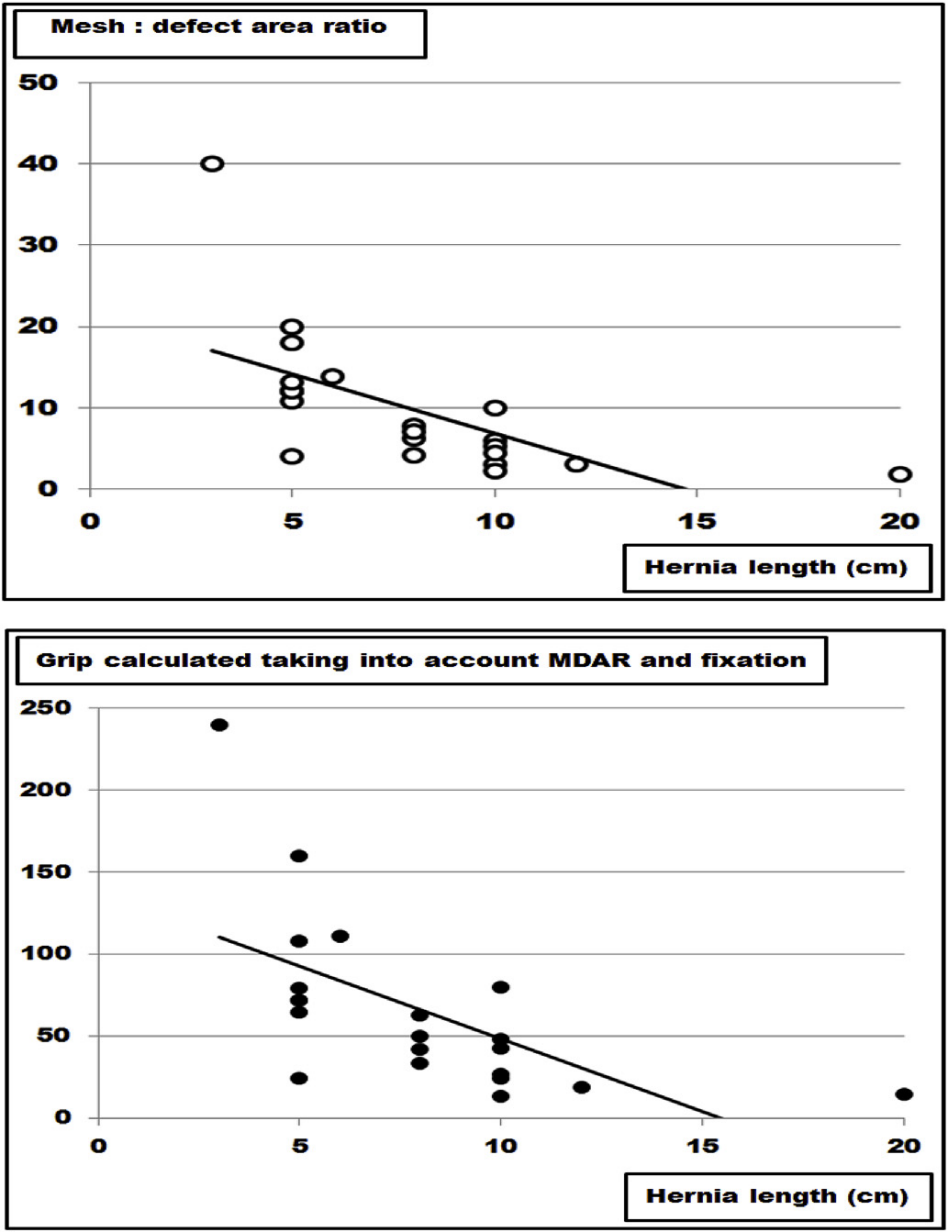
Data from the peritoneal flap hernioplasty as reported earlier [21]. It is obvious that both the mesh: defect area ratio (MDAR) and the grip drop as hernia lengths increase. The line indicates the trend.

This finding is clinically relevant since larger hernia sizes require more advanced procedures such as component separation to implant larger mesh sizes [16,17]. Based on the grip calculations given previously [8], an internet-based application called Stronghold was added to Herniamed, the German-wide registry used for clinical outcome evaluation of ventral hernia repair [8,19,20]. First results show that both MDAR and grip tend to fall with increasing hernia sizes in this clinical registry (Fig. 7). Retrieving the data for the first 20 patients included, a constant drop of the MDAR and of the grip value was noted as hernia size increased (Fig. 7). Searching the literature for a possible relationship between hernia morphology and mesh size or fixation technique, only one manuscript was found which permitted the calculation of the MDAR and the grip related to the length of the incision in the midline [21]. Interestingly, both MDAR and grip dropped with increasing hernia length in the midline in this manuscript (Fig. 8). Due to the low number of patients included in both studies further research is necessary [21]. In Stronghold, the sublay procedure used clinically was adopted to reach higher grip values with larger hernias. It is expected that a more durable repair is observed in Stronghold when using higher grip values for larger hernia sizes.

Rehabilitation programs have been proven to be of benefit for hernia patients [22]. The onset and duration of exercise has to be tailored to the individual patient [23,24]. Stronger repairs, e.g. those with higher grip values, might be exercised earlier. Pain limits postoperative movement. Different fixation devices are followed by different pain levels [25]. It has to be kept in mind that differences in fixation strength as expressed by different grip factors can permit early exercise or limit the biomechanical stability when recommending training (Figs. 2–6). At this point in time, sutures are stronger than Securestraps^®^. Absorbatack^®^ gives less postoperative neuralgia [26] but is to be used with a 1.5 fold increase in fixation points to perform biomechanically stable repairs. Thus, the grip can guide exercise programs following ventral hernia repair. Patients with higher grip values might benefit from an early onset of exercise.

## Provenance and peer review

Not commissioned, externally peer reviewed.

## Ethical approval

The study was conducted using tissue samples derived from animal used for food production.

Animals used for food production are under surveillance according to German law. Use of the tissues was permitted by local authorities in keeping with art. 23 (EG) 1069/2009 with permit DE 08 221 1018 21 according to German law.

## Sources of funding

The work was fully funded by Heidelberger Stiftung Chirurgie grants no. 2016/22, 2017/171, 2018/215 and 2019/288. Material support was supplied by Dahlhausen, Covidien and Ethicon. No conflict of interests occurred since written statements were exchanged for unlimited publication rights concerning each material transfer agreement between the administration of the University and the companies. We acknowledge financial support by Deutsche Forschungsgemeinschaft within the funding programme Open Access Publishing, by the Baden-Württemberg Ministry of Science, Research and the Arts and by Ruprecht-Karls-Universität Heidelberg.

## Author contribution

**The study** was designed by FK, DG, FH and RN.

**The technology** of the bench test was developed by FK, RN and MV.

**Financial and material support** was organized by FK.

**The experiments** were conducted by DG, FH and RN.

**Acquisition and analysis of data** was performed by FK, DG, FH and RN.

**The manuscript** was drafted by FK and carefully reviewed by all authors.

## Conflicts of interest

The work was fully funded by Heidelberger Stiftung Chirurgie grants no. 2016/22, 2017/171, 2018/215 and 2019/288. Material support was supplied by Dahlhausen, Covidien and Ethicon. No conflict of interests occurred since written statements were exchanged for unlimited publication rights concerning each material transfer agreement between the administration of the University and the companies.

## Research registration number

Not applicable since the manuscript designs a bench test.

## Guarantor

Friedrich Kallinowski, Professor of Surgery, Medical Doctor.

Department of General, Visceral and Transplantation Surgery.

University Hospital of Heidelberg.

Im Neuenheimer Feld 110, D-69120 Heidelberg, Germany.

Phone: +49-6221-56-37480.

Fax: +49-6221-56-4637.

Email: friedrich.kallinowski@med.uni-heidelberg.de.

ORCID iD.

Orcid.org/0000-0003-4657-9938.
